# Sex-related differences in the impact of nutritional status on length of hospital stay in atrial fibrillation: a retrospective cohort study

**DOI:** 10.3389/fpubh.2023.1223111

**Published:** 2023-09-06

**Authors:** Adrian Kwaśny, Katarzyna Łokieć, Bartosz Uchmanowicz, Agnieszka Młynarska, Jacek Smereka, Michał Czapla

**Affiliations:** ^1^Institute of Dietetics, The Academy of Business and Health Science, Lodz, Poland; ^2^Department of Propaedeutic of Civilization Diseases, Medical University of Lodz, Lodz, Poland; ^3^Department of Nursing and Obstetrics, Faculty of Health Sciences, Wroclaw Medical University, Wroclaw, Poland; ^4^Department Gerontology and Geriatric Nursing, School of Health Sciences, Medical University of Silesia, Katowice, Poland; ^5^Department of Emergency Medical Service, Wroclaw Medical University, Wroclaw, Poland; ^6^Group of Research in Care (GRUPAC), Faculty of Health Sciences, University of La Rioja, Logroño, Spain; ^7^Institute of Heart Diseases, University Hospital, Wroclaw, Poland

**Keywords:** nutritional status, obesity, malnutrition, sex difference, body mass index, atrial fibrillation

## Abstract

**Background:**

Nutritional status is related to the length of hospitalization of patients with atrial fibrillation (AF). The aim of this study is to assess the prognostic impact of nutritional status and body mass index on length of hospital stay (LOHS) among patients with AF relative to their sex.

**Methods:**

A retrospective analysis of the medical records of 1,342 patients admitted urgently with a diagnosis of AF (ICD10: I48) to the Cardiology Department (University Hospital in Wroclaw, Poland) between January 2017 and June 2021.

**Results:**

In the study group, women were significantly older than men (72.94 ± 9.56 vs. 65.11 ± 12.68, *p* < 0.001). In an unadjusted linear regression model, malnutrition risk was a significant independent predictor of prolonged hospitalization in men (B = 1.95, *p* = 0.003) but not in women. In the age-adjusted linear regression model, malnutrition risk was a significant independent predictor of prolonged hospitalization in men (B = 1.843, *p* = 0.005) but not in women. In the model adjusted for age and comorbidities, malnutrition risk was a significant independent predictor of prolonged hospitalization in men only (B = 1.285, *p* = 0.043). In none of the models was BMI score a predictor of LOHS in either sex.

**Conclusion:**

The risk of malnutrition directly predicts the length of hospital stays in men but not women. The study did not find a relationship between body mass index and length of hospital stay in both women and men.

## 1. Introduction

Atrial fibrillation (AF) is estimated to affect about 2–4% of the adult population, and the incidence is projected to continue to increase up to 4-fold by 2050 (1–3). Both malnutrition and overweight and obesity are challenges to modern public health in developed and developing countries (4). Excess body weight is associated with high cardiovascular risk, risk of prolonged hospitalization and mortality (5–7). Despite knowledge on the subject, malnutrition remains one of the most common causes of death in developing countries. In 70% of cases, nutritional deterioration occurs during hospitalization (8, 9). Several publications indicate an association between AF incidence and overweight and obesity. Studies show a relationship between AF risk and body weight; overweight and underweight were associated with higher arrhythmia events during the follow-up period. It was confirmed that body mass index (BMI), waist circumference, hip circumference and body surface area, among others, were independent predictors of atrial fibrillation (10–12). Thacker et al. observed that higher BMI was an independent factor in arrhythmia progression from paroxysmal or persistent AF to fixed AF, in contrast to other factors (13). Also, a higher BMI score is an independent factor in the progression of arrhythmias from paroxysmal to sustained atrial fibrillation, in contrast to other cardiovascular risk factors (14). Pathak et al. showed that a 10% weight loss in obese patients resulted in a sixfold greater likelihood of maintaining sinus rhythm than patients with no change in body weight values (15). Although some researchers have described a phenomenon occurring among AF patients called the “obesity paradox” concerning deaths from all causes and those from cardiovascular causes, there is an inverse relationship between overweight/obesity and better cardiovascular prognosis at long-term follow-up (16, 17). There are studies showing gender differences in AF (18, 19). However, data on the relationship between nutritional status, gender and length of hospitalization in AF are scarce. This thread has not been sufficiently explored, justifying the need for such observations.

The aim of this study is to assess the prognostic impact of NRS-2002 and body mass index on length of hospital stay (LOHS) among patients with AF relative to their sex.

## 2. Materials and methods

### 2.1. Study design and setting

A retrospective analysis of the medical records of 1,342 patients admitted urgently with a diagnosis of AF (ICD10: I48) to the Cardiology Department (Institute of Heart Diseases, University Hospital in Wroclaw, Poland) between January 2017 and June 2021 was conducted.

### 2.2. Study population and data

Medical records of all patients who met the following inclusion criteria were included in the analysis: emergency admission to the cardiology department for AF (primary reason for hospital admission), BMI and Nutritional Risk Screening 2002 (NRS-2002) score noted in medical records at the time of admission, age ≥18 years old. Finally, data from 1,342 patients were analyzed, such as NRS-2002 score, Body Mass Index (BMI) score, comorbidities: heart failure (HF), chronic kidney disease (CKD), arterial hypertension (HT), diabetes mellitus (DM), thyroid disease (TD), history of cerebral stroke; acute coronary syndrome (ACS) and length of hospital stay (LOHS). Comorbidities and past medical conditions have been recorded by a doctor in the patient’s medical record when the patient is admitted to the hospital.

### 2.3. Tools for assessing nutritional status

Risk of malnutrition was assessed using the screening tools NRS-2002 (20). This tool assesses impaired nutritional status (0–3 points) and severity of disease (0–3 points). If the patient’s age ≥70 years, the patient receives 1 point more. The risk of malnutrition was found when the patient received ≥3 points (20). The WHO criteria for indicating nutritional status are used to classify patients as obese (BMI ≥ 30), pre-obese (BMI 25–29.9), normal body weight (BMI 18.5–24.9), and underweight (BMI < 18.5) (21). Both the NRS-2002 and the BMI score were assessed and recorded in the patient’s medical record by the physician at the time of admission to the hospital.

### 2.4. Ethical considerations

The study was conducted following the principles of the Declaration of Helsinki and approved by the independent Bioethics Committee of Wroclaw Medical University, protocol no. KB-837/2022. The study followed the STROBE guidelines (Strengthening the Reporting of Observational Studies in Epidemiology).

### 2.5. Statistical analysis

Distributions of quantitative variables were summarized with mean, standard deviation, median and quartiles. In contrast, distributions of qualitative variables were summarized with the number and percent of occurrence for each value. Chi-squared test (with Yates’ correction for 2 × 2 tables) was used to compare qualitative variables among groups. In the case of low values in contingency tables, Fisher’s exact test was used instead. Mann–Whitney test was used to compare quantitative variables between two groups. Logistic regression was used to analyze the impact of quantitative variables on dichotomous outcomes. All clinical variables recorded in medical records at hospital admission were used to construct a multivariate model. Odds ratios (OR) with 95% confidence intervals were shown. The significance level for all statistical tests was set to 0.05. R 4.2.2. was used for computations.

## 3. Results

### 3.1. Comparison of patient characteristics by sex

All 1,342 patients were included in the analysis. In the first step, a comparison was made concerning gender. A comparison of the groups by gender is shown in Table 1. Women were significantly older than men (72.94 ± 9.56 vs. 65.11 ± 12.68, *p* < 0.001). Women were also significantly more likely than men to suffer from CKD, thyroid disease and stroke history. This group also had a higher risk of malnutrition (11.09% vs. 3.99%, *p* < 0.001).

**Table 1:** Comparison of patient characteristics by sex.

Parameter		Female (*N* = 568)	Male (*N* = 774)	Total (*N* = 1,342)	*p*
Age [years]	Mean (SD)	72.94 (9.56)	65.11 (12.98)	68.42 (12.28)	<0.001*
	Median (quartiles)	72 (68–80)	67 (59–73)	70 (63–76)	
	Range	31–94	19–95	19–95	
Type of AF	Paroxysmal	236 (41.55%)	281 (36.30%)	517 (38.52%)	0.04*
	Persistent	227 (39.96%)	363 (46.90%)	590 (43.96%)	
	Permanent	105 (18.49%)	130 (16.80%)	235 (17.51%)	
HF	No	449 (79.05%)	639 (82.56%)	1,088 (81.07%)	0.121
	Yes	119 (20.95%)	135 (17.44%)	254 (18.93%)	
DM	No	449 (79.05%)	613 (79.20%)	1,062 (79.14%)	1
	Yes	119 (20.95%)	161 (20.80%)	280 (20.86%)	
CKD	No	474 (83.45%)	688 (88.89%)	1,162 (86.59%)	0.005*
	Yes	94 (16.55%)	86 (11.11%)	180 (13.41%)	
CS	No	491 (86.44%)	697 (90.05%)	1,188 (88.52%)	0.05*
	Yes	77 (13.56%)	77 (9.95%)	154 (11.48%)	
HT	No	233 (41.02%)	346 (44.70%)	579 (43.14%)	0.197
	Yes	335 (58.98%)	428 (55.30%)	763 (56.86%)	
ACS	No	502 (88.38%)	699 (90.31%)	1,201 (89.49%)	0.294
	Yes	66 (11.62%)	75 (9.69%)	141 (10.51%)	
TD	No	411 (72.36%)	682 (88.11%)	1,093 (81.45%)	<0.001*
	Yes	157 (27.64%)	92 (11.89%)	249 (18.55%)	
BMI	18.5–24.9	154 (27.11%)	182 (23.51%)	336 (25.04%)	0.124
	<18.5	2 (0.35%)	3 (0.39%)	5 (0.37%)	
	25.0–29.9	193 (33.98%)	310 (40.05%)	503 (37.48%)	
	≥30	219 (38.56%)	279 (36.05%)	498 (37.11%)	
NRS-2002	<3	460 (80.99%)	652 (84.24%)	1,112 (82.86%)	<0.001*
	≥3	63 (11.09%)	30 (3.88%)	93 (6.93%)	
	Unknown	45 (7.92%)	92 (11.89%)	137 (10.21%)	
LOHS [days]	Mean (SD)	4.41 (3.16)	4.31 (3.42)	4.35 (3.31)	0.234
	Median (quartiles)	4 (3–6)	3 (2–5)	3 (2–5.75)	
	Range	1–26	1–34	1–34	
BMI [kg/m^2^]	Mean (SD)	28.95 (5.55)	28.67 (4.58)	28.79 (5.01)	0.654
	Median (quartiles)	28.3 (24.6–32.8)	28.1 (25.2–31.6)	28.3 (25–32)	
	Range	18.5–48.9	18.5–56.8	18.5–56.8	

*p* – qualitative variables: chi-squared or Fisher’s exact test. Quantitative variables: Mann–Whitney test. *Statistically significant (*p* < 0.05). *n*, number of participants; AF, atrial fibrillation; HF, heart failure; CKD, chronic kidney disease; HT, arterial hypertension; DM, diabetes mellitus; CS, cerebral stroke; ACS, acute coronary syndrome; TD, thyroid disease; BMI, body mass index; NRS-2002, Nutritional Risk Score; LOHS, length of hospital stay.

### 3.2. Group comparison relative to obesity

Patients of each gender were divided into two groups according to WHO criteria: obese (BMI ≥ 30) and non-obese (BMI < 30). Women with obesity were significantly more likely to be younger and have DM compared to women in the non-obese group. Women with obesity were significantly less likely to have CKD, CS and less likely to have malnutrition risk, according to NRS-2002, compared to the obese group. Obese men were significantly younger than non-obese men. Obese men were less often at risk in malnutrition (Table 2).

**Table 2:** Comparison of patient characteristics by absence or presence of obesity.

Parameter		Female (*N* = 568)			Male (*N* = 794)		
		Obese (*N* = 219)	Non-obese (*N* = 349)	*p*	Obese (*N* = 279)	Non-obese (*N* = 495)	*p*
Age [years]	Mean (SD)	70.13 (7.24)	74.7 (10.38)	<0.001*	63.78 (10.39)	65.85 (14.19)	0.001*
	Median (quartiles)	70 (66.5–74)	74 (68–83)		66 (58–70)	68 (60–74)	
	Range	46–91	31–94		35–93	19–95	
Type of AF	Paroxysmal	79 (36.07%)	157 (44.99%)	<0.001*	77 (27.60%)	204 (41.21%)	<0.001*
	Persistent	113 (51.60%)	114 (32.66%)		163 (58.42%)	200 (40.40%)	
	Permanent	27 (12.33%)	78 (22.35%)		39 (13.98%)	91 (18.38%)	
HF	No	167 (76.26%)	282 (80.80%)	0.234	220 (78.85%)	419 (84.65%)	0.052
	Yes	52 (23.74%)	67 (19.20%)		59 (21.15%)	76 (15.35%)	
DM	No	160 (73.06%)	289 (82.81%)	0.008*	213 (76.34%)	400 (80.81%)	0.169
	Yes	59 (26.94%)	60 (17.19%)		66 (23.66%)	95 (19.19%)	
CKD	No	193 (88.13%)	281 (80.52%)	0.024*	248 (88.89%)	440 (88.89%)	1
	Yes	26 (11.87%)	68 (19.48%)		31 (11.11%)	55 (11.11%)	
CS	No	200 (91.32%)	291 (83.38%)	0.01*	254 (91.04%)	443 (89.49%)	0.573
	Yes	19 (8.68%)	58 (16.62%)		25 (8.96%)	52 (10.51%)	
HT	No	79 (36.07%)	154 (44.13%)	0.07	117 (41.94%)	229 (46.26%)	0.277
	Yes	140 (63.93%)	195 (55.87%)		162 (58.06%)	266 (53.74%)	
ACS	No	197 (89.95%)	305 (87.39%)	0.428	250 (89.61%)	449 (90.71%)	0.711
	Yes	22 (10.05%)	44 (12.61%)		29 (10.39%)	46 (9.29%)	
TD	No	165 (75.34%)	246 (70.49%)	0.245	248 (88.89%)	434 (87.68%)	0.701
	Yes	54 (24.66%)	103 (29.51%)		31 (11.11%)	61 (12.32%)	
NRS-2002	<3	187 (85.39%)	273 (78.22%)	<0.001*	236 (84.59%)	416 (84.04%)	0.006*
	≥3	4 (1.83%)	59 (16.91%)		3 (1.08%)	27 (5.45%)	
LOHS [days]	Mean (SD)	4.25 (2.98)	4.52 (3.27)	0.527	4.13 (2.87)	4.41 (3.7)	0.953
	Median (quartiles)	4 (2.5–6)	4 (3–6)		3 (2–5)	3 (2–5)	
	Range	1–24	1–26		1–22	1–34	

*p* – qualitative variables: chi-squared or Fisher’s exact test. Quantitative variables: Mann–Whitney test. *Statistically significant (*p* < 0.05). *n*, number of participants; AF, atrial fibrillation; HF, heart failure; CKD, chronic kidney disease; HT, arterial hypertension; DM, diabetes mellitus; CS, cerebral stroke; ACS, acute coronary syndrome; TD, thyroid disease; TG, triglycerides; LDL, low-density lipoprotein; HDL, high-density lipoprotein; TC, total cholesterol; hsCRP, high-sensitivity C-reactive protein; K, potassium; Na, sodium; BMI, body mass index; NRS-2002, Nutritional Risk Score; LOHS, length of hospital stay.

### 3.3. Group comparison against malnutrition risk

Women at risk for malnutrition were significantly older. They also were more likely to have CKD and less likely to have HT and TD, and had a lower BMI compared to the group without malnutrition risk. Men at risk of malnutrition were less likely to have diseases such as DM and HT. Men in this group also lower BMIs than men at no malnutrition risk (Table 3).

**Table 3:** Group comparison concerning malnutrition risk.

Parameter		Female (*N* = 523)			Male (*N* = 682)		
		NRS ≥ 3 (*N* = 63)	NRS < 3 (*N* = 460)	*p*	NRS ≥ 3 (*N* = 30)	NRS < 3 (*N* = 652)	*p*
Age [years]	Mean (SD)	76.59 (10.78)	72.65 (9.52)	<0.001*	69.77 (17.39)	65.18 (12.89)	0.051
	Median (quartiles)	78 (72–84)	72 (67–80)		72.5 (64–83)	67 (59–73)	
	Range	31–92	33–94		26–95	19–95	
Type of AF	Paroxysmal	26 (41.27%)	194 (42.17%)	0.985	9 (30.00%)	240 (36.81%)	0.197
	Persistent	24 (38.10%)	175 (38.04%)		12 (40.00%)	300 (46.01%)	
	Permanent	13 (20.63%)	91 (19.78%)		9 (30.00%)	112 (17.18%)	
HF	No	50 (79.37%)	363 (78.91%)	1	26 (86.67%)	538 (82.52%)	0.733
	Yes	13 (20.63%)	97 (21.09%)		4 (13.33%)	114 (17.48%)	
DM	No	51 (80.95%)	369 (80.22%)	1	29 (96.67%)	514 (78.83%)	0.032*
	Yes	12 (19.05%)	91 (19.78%)		1 (3.33%)	138 (21.17%)	
CKD	No	43 (68.25%)	390 (84.78%)	0.002*	24 (80.00%)	577 (88.50%)	0.154
	Yes	20 (31.75%)	70 (15.22%)		6 (20.00%)	75 (11.50%)	
CS	No	53 (84.13%)	396 (86.09%)	0.821	27 (90.00%)	585 (89.72%)	1
	Yes	10 (15.87%)	64 (13.91%)		3 (10.00%)	67 (10.28%)	
HT	No	35 (55.56%)	184 (40.00%)	0.027*	23 (76.67%)	281 (43.10%)	0.001*
	Yes	28 (44.44%)	276 (60.00%)		7 (23.33%)	371 (56.90%)	
ACS	No	53 (84.13%)	408 (88.70%)	0.399	30 (100.00%)	582 (89.26%)	0.062
	Yes	10 (15.87%)	52 (11.30%)		0 (0.00%)	70 (10.74%)	
TD	No	53 (84.13%)	323 (70.22%)	0.031*	27 (90.00%)	571 (87.58%)	1
	Yes	10 (15.87%)	137 (29.78%)		3 (10.00%)	81 (12.42%)	
BMI	Underweight	2 (3.17%)	0 (0.00%)	<0.001*	0 (0.00%)	3 (0.46%)	<0.001*
	Normal	36 (57.14%)	116 (25.22%)		19 (63.33%)	152 (23.31%)	
	Overweight	21 (33.33%)	157 (34.13%)		8 (26.67%)	261 (40.03%)	
	Obesity	4 (6.35%)	187 (40.65%)		3 (10.00%)	236 (36.20%)	
LOHS [days]	Mean (SD)	4.48 (2.84)	4.5 (3.22)	0.802	6.4 (5.85)	4.33 (3.27)	0.051
	Median (quartiles)	4 (2–6.5)	4 (3–6)		4 (3–7)	3 (3–5)	
	Range	1–13	1–26		1–23	1–34	

*p* – qualitative variables: chi-squared or Fisher’s exact test. Quantitative variables: Mann–Whitney test. *Statistically significant (*p* < 0.05). *n*, number of participants; AF, atrial fibrillation; HF, heart failure; CKD, chronic kidney disease; HT, arterial hypertension; DM, diabetes mellitus; CS, cerebral stroke; ACS, acute coronary syndrome; TD, thyroid disease; BMI, body mass index; NRS-2002, Nutritional Risk Score; LOHS, length of hospital stay.

### 3.4. Effect of BMI and NRS-2002 on LOHS – unadjusted and adjusted for age

For women, a multivariate linear regression model showed that none of the analyzed characteristics was a significant independent predictor of hospitalization length. However, for men, a significant independent predictor of prolonged hospitalization was the risk of malnutrition (B = 1.95; *p* = 0.003), which prolonged hospitalization by an average of almost 1.95 days (Table 4). In an age-adjusted linear regression model, this proved to be a significant independent predictor of LOHS in both women (B = 0.072, *p* < 0.001) and men (B = 0.035, *p* = 0.001). In addition, in men, the risk of malnutrition remained a significant independent predictor (B = 1.843, *p* = 0.005), which on average prolonged hospitalization by 1.843 days (Table 4).

**Table 4:** Effect of BMI and NRS-2002 on LOHS – unadjusted and adjusted for age.

Unadjusted model	Trait		B	95%CI		*p*
**Female**	BMI	18.5–24.9	ref.			
		<18.5	−1.976	−6.467	2.515	0.389
		25.0–29.9	0.203	−0.494	0.9	0.569
		≥30	−0.072	−0.776	0.632	0.841
	NRS-2002	<3	ref.			
		≥3	0.012	−0.871	0.894	0.98
**Male**	BMI	18.5–24.9	ref.			
		<18.5	−1.561	−5.468	2.346	0.434
		25.0–29.9	−0.318	−0.982	0.346	0.348
		≥30	−0.263	−0.947	0.42	0.45
	NRS-2002	<3	ref.			
		≥3	1.95	0.674	3.226	0.003*

Adjusted for age	Trait		B	95%CI		*p*
**Female**	BMI	18.5–24.9	ref.			
		<18.5	−0.59	−5.017	3.837	0.794
		25.0–29.9	0.167	−0.515	0.849	0.632
		≥30	0.228	−0.471	0.927	0.523
	NRS-2002	<3	ref.			
		≥3	−0.213	−1.081	0.656	0.631
	Age	[years]	0.072	0.043	0.1	<0.001*
**Male**	BMI	18.5–24.9	ref.			
		<18.5	−1.842	−5.722	2.037	0.352
		25.0–29.9	−0.218	−0.879	0.443	0.518
		≥30	−0.115	−0.798	0.568	0.742
	NRS-2002	<3	ref.			
		≥3	1.843	0.576	03.lis	0.005*
	Age	[years]	0.035	0.015	0.054	0.001*

B, unstandardized regression coefficient; *p*, multiple linear regression. *Statistically significant (*p* < 0.05). BMI, Body Mass Index; NRS-2002, Nutrition Risk Screening.

### 3.5. Impact of BMI and NRS on length of hospitalization – adjusted for comorbidities

For female patients, a multivariate linear regression model showed that significant independent predictors of length of hospitalization are age (B = 0.075), persistent AF (B = 0.717), and HT (B = −0.751). In male patients, independent predictors of length of hospitalization are age (B = 0.029), persistent AF (B = −0.612), permanent AF (B = 1.217), history of stroke (B = −1.598), and HT (B = −0.979). Still, despite the addition of comorbidities to the model, the risk of malnutrition was a significant independent factor affecting the length of hospitalization (B = 1.285, *p* = 0.043) in men (Table 5).

**Table 5:** Effect of BMI and NRS-2002 on LOHS in men and women – adjusted model.

	Trait		B	95%CI		*p*
Female	Age	[years]	0.075	0.045	0.105	<0.001*
	Type of AF	Paroxysmal	ref.			
		Persistent	0.717	0.119	1.315	0.019*
		Permanent	0.028	−0.753	0.808	0.945
	HF	No	ref.			
		Yes	0.434	−0.286	1.153	0.238
	DM	No	ref.			
		Yes	−0.043	−0.756	0.67	0.905
	CKD	No	ref.			
		Yes	−0.474	−1.266	0.317	0.241
	CS	No	ref.			
		Yes	−0.379	−1.213	0.456	0.374
	HT	No	ref.			
		Yes	−0.751	−1.357	−0.145	0.016*
	ACS	No	ref.			
		Yes	−0.692	−1.56	0.176	0.119
	TD	No	ref.			
		Yes	−0.154	−0.751	0.443	0.613
	BMI	18.5–24.9	ref.			
		<18.5	−0.917	−5.286	3.453	0.681
		25.0–29.9	0.047	−0.636	0.731	0.893
		≥30	0.004	−0.706	0.714	0.992
	NRS-2002	<3	ref.			
		≥3	−0.312	−1.183	0.559	0.483
Male	Age	[years]	0.029	0.008	0.05	0.007*
	Type of AF	Paroxysmal	ref.			
		Persistent	−0.612	−1.185	−0.039	0.037*
		Permanent	1.217	0.42	2.014	0.003*
	HF	No	ref.			
		Yes	0.383	−0.323	1.089	0.288
	DM	No	ref.			
		Yes	−0.63	−1.309	0.049	0.07
	CKD	No	ref.			
		Yes	0.764	−0.06	1.587	0.069
	CS	No	ref.			
		Yes	−1.598	−2.459	−0.737	<0.001*
	HT	No	ref.			
		Yes	−0.979	−1.544	−0.414	0.001*
	ACS	No	ref.			
		Yes	0.154	−0.722	1.03	0.731
	TD	No	ref.			
		Yes	−0.181	−0.935	0.573	0.638
	BMI	18.5–24.9	ref.			
		<18.5	−2.594	−6.37	1.182	0.179
		25.0–29.9	−0.001	−0.652	0.649	0.997
		≥30	0.138	−0.539	0.816	0.689
	NRS-2002	<3	ref.			
		≥3	1.285	0.042	2.529	0.043*

B, unstandardized regression coefficient; *p*, multiple linear regression. *Statistically significant (*p* < 0.05). *n*, number of participants; AF, atrial fibrillation; HF, heart failure; CKD, chronic kidney disease; HT, arterial hypertension; DM, diabetes mellitus; CS, cerebral stroke; ACS, acute coronary syndrome; TD, thyroid disease; BMI, body mass index; NRS-2002, Nutritional Risk Score.

## 4. Discussion

The impact of nutritional status on CVD is widely reported in the scientific literature. Its effects can range from the risk of cardiovascular events, presentation of symptoms, condition treatment methods, length of hospitalization and influence patient prognosis. The association of obesity with AF and the effect of weight reduction on its course is well known (22). It is also known that being underweight can be an independent risk factor for AF, and the association of BMI with AF risk takes a “U” shape (23). Scientific reports are also increasingly pointing out the gender differences present in atrial fibrillation (24). However, to the best of our knowledge, this study is one of the few to evaluate gender differences in the effect of nutritional status on the length of hospitalization in patients with AF, highlighting the complexity of this problem.

In the study, a multivariate linear regression model showed that malnutrition risk, as determined by the NRS-2002 scale, was a significant independent predictor of prolonged LOHS in men (B = 1.285, *p* = 0.005). No such effect was demonstrated in women. We also noted no effect of BMI score on LOHS for either sex. A study by Cheng et al. confirmed the impact of malnutrition on clinical outcomes, which showed that moderate to severe malnutrition is an independent predictor of adverse prognosis among older adult patients with non-valvular AF (25). The impact on LOHS was also evaluated in the work of Alturi et al., where it was shown that protein-calorie malnutrition in patients with AF could prolong hospital stay by 2.76 days (26). When comparing the groups in relation to the risk of malnutrition, statistically significant differences in the length of hospitalization in both men and women were not registered. However, the length of hospitalization is prolonged in the group of men with NRS ≥ 3 (6.4 vs. 4.33), which, although not statistically significant, may be clinically relevant and affect the total cost of treatment.

The risk of malnutrition can be studied using several different tools. Zhu et al. evaluated the effect of malnutrition assessed by the nutritional status score (CONUT score) and geriatric nutritional risk index (GNRI) on AF recurrence in patients after ablation procedures. They found that malnourished patients were more likely to experience AF recurrence (27). Malnutrition can also affect the increased risk of complications. Kim et al. showed that it increases the risk of complications in AF patients undergoing catheter ablation. The overall complication rate was more marked among malnourished women (7.1%) than malnourished men (3.7%) (28). Monitoring patients’ nutritional status is essential to the medical care process, as it can deteriorate during a hospital stay (29). The assessment should be performed at the time of admission to the hospital and during hospitalization. This is because it has been shown that a drop in category on the Subjective Global Assessment (SGA) or significant weight loss during the first week of hospitalization may be associated with a greater likelihood of a longer hospital stay (30).

In our study, only males had malnutrition risk according to NRS-2002 as a significant independent predictor of LOHS. Although this hypothesis requires further research, it may be influenced by different body fat content relative to gender. It is indicated that with similar BMI, the body fat percentage in men is lower than in women (31). In our study, we also found no effect of BMI on LOHS in either sex, which seems to confirm the lack of reflection of body composition in the BMI parameter. This is because it does not consider body fat, muscle mass or water content but only the patient’s weight-to-height ratio.

Researchers identify multiple determinants of prolonged hospital stay for patients with AF. Independent predictors of LOHS include acute coronary syndromes, acute decompensated heart failure, heart failure with reduced ejection fraction, and elevated NT-proBNP levels (32). Sex differences in AF are related to comorbidities, the influence of sex hormones, differences in electrophysiology, endothelial dysfunction, and pro-inflammatory signalling, among other factors (33). Researchers indicate that women with AF have a larger left atrial diameter, which affects their mortality (34), prolonged hospitalization time compared to men after ablation (35), and a higher risk of AF recurrence after radiofrequency catheter ablation (35). Women with AF also report poorer overall quality of life (36). Although in our study, BMI results were not a factor in the length of hospitalization, it should be noted that many authors show a positive association between the occurrence of AF and obesity, overweight and underweight (37–39). Also, in the study we conducted, there were no deaths; however, it is worth noting that increasingly researchers are pointing to gender differences in the incidence of mortality and the course of atrial fibrillation (40, 41). Renoux et al. showed that AF mortality was higher in males (10.0, 95% CI 9.8 to 10.1) than in females (8.5, 95% CI 8.3 to 8.6) (40). Our findings of gender differences in the effects of BMI and NRS on LOHS in patients with AF justify the need for further prospective studies in this area, highlighting the complexity of factors affecting the length of hospitalization.

### 4.1. Study limitation

This study had several limitations. The percentage of male patients at risk for malnutrition was low at 3.88%. In addition, due to the study’s retrospective nature, among other factors, patients’ body composition was not analyzed by electrical bioimpedance or anthropometric measurements were not taken. The patient’s body composition was not assessed in the present study, only the NRS-2002 score and BMI. Anthropometric differences between genders may affect prognosis, which may have been a limitation of this study. Due to restrictions on access to patients’ data under Polish law, the long-term survival of patients with AF could not be assessed.

## 5. Conclusion

The risk of malnutrition according to the NRS-2002 directly predicts the length of hospital stays in men but not women. The study did not find a relationship between body mass index and length of hospital stay in both women and men. Because the number of participants were at risk of malnutrition, these results should be interpreted within the context of each patient. Additional independent predictors of length of hospitalization for female patients independent predictors of length of hospitalization are age, persistent AF, hypertension and in male patient’s age, persistent AF, permanent AF, history of stroke and hypertension. Undoubtedly, the impact of NRS-2002 and BMI results in patients hospitalized in the cardiology department due to atrial fibrillation relative to sex requires further investigation.

## Data availability statement

The original contributions presented in this study are included in the article, further inquiries can be directed to the corresponding author.

## Author contributions

AK and MC: conceptualization, methodology, validation, formal analysis, resources, writing-original draft preparation, and writing-review and editing. AK and KŁ: software. MC, BU, AM, KŁ, and JS: investigation. AK: data curation. AK and AM: visualization. MC: supervision. KŁ: project administration. JS: funding acquisition. All authors contributed to the article and approved the submitted version.

## Funding

The study was funded by the Ministry of Science and Higher Education of Poland under the statutory grant of the Wroclaw Medical University (SUBZ.E250.23.020).

## Conflict of interest

The authors declare that the research was conducted in the absence of any commercial or financial relationships that could be construed as a potential conflict of interest.

## Publisher’s note

All claims expressed in this article are solely those of the authors and do not necessarily represent those of their affiliated organizations, or those of the publisher, the editors and the reviewers. Any product that may be evaluated in this article, or claim that may be made by its manufacturer, is not guaranteed or endorsed by the publisher.
